# Exercising Hope in Palliative Care Is Celebrating Spirituality: Lessons and Challenges in Times of Pandemic

**DOI:** 10.3389/fpsyg.2022.933767

**Published:** 2022-06-29

**Authors:** Carlos Laranjeira, Filipa Baptista Peixoto Befecadu, Maria Goreti Da Rocha Rodrigues, Philip Larkin, Sophie Pautex, Maria Anjos Dixe, Ana Querido

**Affiliations:** ^1^School of Health Sciences of Polytechnic of Leiria, Leiria, Portugal; ^2^Centre for Innovative Care and Health Technology (ciTechCare), Polytechnic of Leiria, Leiria, Portugal; ^3^Research in Education and Community Intervention (RECI I&D), Piaget Institute, Viseu, Portugal; ^4^Palliative and Supportive Care Service and Institute of Higher Education and Research in Healthcare, Lausanne University Hospital, University of Lausanne, Lausanne, Switzerland; ^5^HESAV School of Health Sciences, HES-SO University of Applied Sciences and Arts Western, Lausanne, Switzerland; ^6^High School of Health (HEdS), Geneva, Switzerland; ^7^Department of Readaptation and Geriatrics, Palliative Medicine Division, University Hospital Geneva and University of Geneva, Geneva, Switzerland; ^8^Center for Health Technology and Services Research (CINTESIS), NursID, University of Porto, Porto, Portugal

**Keywords:** hope, spirituality, positive psychology, palliative care, healthcare professionals, COVID-19 pandemic, continuing education

## Introduction

Times of crisis are times of suffering and pain and opportunities for transformation. Beyond the current debate on measures to combat coronavirus disease 2019 (COVID-19), one must consider two consequences of the pandemic that can be opportunities to foster spiritual well-being (Stilos et al., 2021) and exercise hope in palliative care.

First, the pandemic made us remember our human fragility (Bunkers, 2020; Lozupone et al., 2020). The trail of death, fear, and social paralysis brought on by severe acute respiratory syndrome coronavirus 2 (SARS-CoV-2) has imposed a heavy blow against the dictatorship of human narcissism and exposed our human frailty before an invisible and insidious threat that separates us from our loved ones. In this perspective, there are paths of spirituality leading deep into ourselves, introspectively, in search of meaning and connection with others, toward transcendence and the Divine (Steinhauser et al., 2017; Worth and Smith, 2021). Greater spiritual well-being is closely tied with a sense of dignity and self-worth, of having lived a full and fulfilling life (Roman et al., 2020). Evidence indicates that spirituality has an essential role in mental and physical health, serving as a protective element during psychological adjustment to unpleasant situations (Niemiec et al., 2020).

Second, in addition to our fragility, this new reality reminds us that we are mutually dependent beings. No one can take care of themselves alone in the face of disease, bringing us to the edge of our finitude. We are social beings, created for solidarity, connection, and affection. We could use these unprecedented times to experience deeper connectivity with what and who matters (Long et al., 2022).

Given the psychological consequences of COVID-19-related stressors, positive and hopeful thinking may be a beneficial resource that allows individuals to maintain or regain their well-being (Hamouche, 2020; Büssing et al., 2021; Counted et al., 2022). Fostering education on the pedagogy of hope and its ties with spiritual well-being (Jaiswal et al., 2014) can contribute to education for alterity. In alterity, commitment to hope implies a pledge to create conditions and dynamics of hope for others. Only then can there be an effective change in attitudes and the creation of civic awareness of the responsibility in constructing a society of hope. Addressing spirituality and hope may improve knowledge of healthy human functioning and flourishing mental health, as well as represent “life worth living” in general (Rego and Nunes, 2019). Spirituality and character strengths, like hope, can both promote greater wholeness in one's psycho-spiritual journey and contribute to the communal good (Niemiec et al., 2020).

Herein, we intend to underline and reflect on hope as a practice of spiritual care in palliative care, especially because hope is, itself, a pedagogy of life that helps one fulfill themselves as a person. We highlight the exercise of hope in end-of-life contexts, particularly in the training of palliative care professionals.

## Promoting Hope As Spiritual Care

According to Leget (2018), spiritual needs can be apparent, implicit, or even buried, and they can be interwoven with other sorts of needs. This definition is based on three basic notions: *the sacred or transcendent; a relationship with the sacred; and the search for ultimate meaning or purpose* (Peterson and Seligman, 2004; Mayseless and Russo-Netzer, 2017; Niemiec et al., 2020).

By placing a relatively new emphasis on character strengths and virtues, positive psychology can help position spirituality as a human character strength. Following the Platonic and Aristotelian traditions, the Values-In-Action (VIA) framework considers transcendence as a virtue, which includes character strengths, such as spirituality, gratitude, and hope (Peterson and Seligman, 2004; Kor et al., 2019).

In this context, hope is conceptualized as a positive character trait that enables individuals to thrive and flourish (Kor et al., 2019; Niemiec et al., 2020), based not on external results (such as expectation), but on one's personal fulfillment (a radical change in the human condition) (Pinsent, 2020). Thus, hope is internal energy forever growing and enabling one to break down walls and obstacles (Guedes et al., 2021). The experience of hope transmits peace and security to the present and, thus, gives strength to walk with confidence toward the future.

In this sense, a pedagogy of hope should be present in the loving, supportive, encouraging, and activating presence of those who work in palliative care. Every therapeutic intervention should be committed to hope, translated, and materialized in a personal attitude and ability to help others look at reality differently, with neither a facile optimism nor a destructive pessimism, but a realistic, active, questioning hope (Olsman, 2022).

Hope is a product of human action, but also an ontological necessity (Torres-Olave, 2021). That is why, in desperate situations (e.g., spiritual anguish), hope appears as the only source of salvation, leading people to look ahead and search for new ways to solve difficulties, especially when the remedy is complicated or cannot be found in science, technology, and social development. This is the case of terminal illnesses that are both desperate and intrusive. When faced with such situations, many people give in to despair and disillusionment, as evidenced by the increase in suicidality (Salamanca-Balen et al., 2021), and experience anxiety and depression (Lee et al., 2022), while others continue to nourish the hope that better days will come, even invoking divine protection, thus, envisioning improvements in health or family life.

## Why We Wait: Fostering Hope in Palliative Care

Curiously, in the face of adverse socio-economic and cultural circumstances (which mainly victimize the most vulnerable), hope appears as the main and last stronghold a person can cling to, even if resigned to their fate (Javier-Aliaga et al., 2022). The same is true of victims of incurable diseases or other situations, for which all human, scientific, and technological capacities seem exhausted, but who nevertheless claim to have an unshakable faith and hope. Several studies support the existence of spiritual distress in patients who are seriously ill that require coping strategies (e.g., hope) to overcome suffering (Kondejewski and Sinclair, 2018; Martins and Caldeira, 2018). Why do these people continue to wait, despite all their setbacks?

Hope is present in every part of our lives, as a search for the meaning of life, particularly in times of suffering. Viktor Frankl's book *Man's Search for Meaning* drew attention to what he called “the existential vacuum” and how human beings are unable to develop in an unfavorable environment, without meaning, order, trust, and stability (Schimmoeller and Rothhaar, 2021). Meaning is an essential element of a human being throughout the construction of their life (Feldman et al., 2018). We are beings devoted to the search for meaning. Biologically, our nervous system is structured such that external stimuli are automatically organized by the brain into internally meaningful structures (Genon et al., 2018). Psychologically, hope is a response of trust, which, at the same time, allows us to find the meaning of difficulties and trials in life, including the most serious. However, some situations exceed our abilities and control, when the presence of meaning in life is beyond our control. This is when hope comes into play when it guarantees the presence of meaning in life and promotes life satisfaction (Karata et al., 2021).

Given the positive effects of hope in palliative care, various psychospiritual and clinical strategies to foster hope or diminish hopelessness have been documented in the palliative care literature (Laranjeira et al., 2020). Herth (2000), for example, designed a nursing intervention program to enhance hope and quality of life in a group of cancer patients. Duggleby and Williams (2010) developed a group intervention program designed to help palliative caregivers endure suffering by living with hope. Similarly, dignity therapy (a brief psychological intervention based on scientific understandings of dignity toward the end-of-life) has been tried in a diverse group of patients in palliative care (Salamanca-Balen et al., 2021). Some strategies have been intended primarily to improve other outcomes, with hope or hopelessness measured as a secondary outcome. Meaning-centered psychotherapy studies, for example, often assess spiritual well-being and quality of life as key outcomes and hopelessness as a secondary outcome (Breitbart et al., 2012).

A recent meta-analysis found that hope-fostering interventions improved spirituality and lowered depression considerably (Salamanca-Balen et al., 2021). Hope and spirituality may be connected, which would explain why these interventions affect both outcomes. Moreover, there is evidence that multi-component interventions (such as early palliative care, which incorporates particular medical, social, and psychological components) can be beneficial in enhancing the quality of life, lowering depression, and increasing survival (Temel et al., 2010). Thus, hope is not just a virtue, a source of energy that allows us to face and overcome barriers and difficulties, but also a fundamental dynamic of living, enabling one to face life with a different perspective and renewed meaning, even when none seems to exist (Colla et al., 2022). While a lack of judgment or critical thinking about one's spiritual beliefs can result in a narrow and selfish worldview that becomes dogmatic and rigid, too much hope may cause a person to only see the positive aspects of their spirituality and ignore the negative aspects or limitations (Niemiec et al., 2020). The simplistic concept of “good” spirituality may lead researchers to overlook the potentially damaging aspects of spiritual life. There are many examples of those seeking intimacy with God *via* generosity and compassion, who also employ intense self-punishing asceticism to attain their sacred aspirations. By definition, ignoring the dark side of spirituality results in an incomplete or inaccurate depiction of the phenomena (Paloutzian and Park, 2013).

Religious and spiritual struggles may compromise the well-being and positive mental states and be related to anxiety or depression in those receiving palliative care (Damen et al., 2021). The most common spiritual struggles in end-of-life care include the following: “anger or disappointment with God, feeling abandoned, or unloved by God; tensions and guilt about not living up to one’s higher standards and wrestling with attempts to follow moral principles; and concerns that life may not matter, and questions about whether one’s own life has a deeper meaning” (Pargament and Exline, 2020, p.1). To properly apply spiritual care in palliative care at this stage, healthcare providers must build their spiritual competency *via* education and self-reflection (Gijsberts et al., 2019).

## Continuing Education Program For Healthcare Providers in Palliative Care: Some Recommendations

As mentioned, understanding personhood cannot dispense with analyzing the conditions that make being a person possible. Personhood is inseparable from living with dignity and, consequently, from the common good that makes it possible. Hence, the mandatory inclusion of the approach of spirituality and hope when training professionals who face end-of-life situations is part of a more humanized and patient-centered model of care (García-Navarro et al., 2021). Regrettably, these professions are not sufficiently trained and prepared to deal with spirituality and interventions related to finitude (Wu et al., 2016; Oliveira et al., 2021).

As part of an international consortium between higher education institutions in Portugal (Polytechnic of Leiria) and Universities of Applied Sciences and Arts Western Switzerland-HES-SO (Lausanne and Geneva), a continuing education program on spirituality was designed for palliative care professionals. This proposal aims to promote the experience of hope in palliative care, thus, making it possible to: understand that hope is a humanizing virtue that generates dignity, love, and meaning in life and to understand hope as a force and dynamism that spurs an active commitment to the other and compassion in care.

Therefore, this type of continuous training (Table 1) will provide professionals with a coherent and authentic vision of hope in contexts where death and dying happen persistently. Only in this way will they understand hope as something to be hoped for and believed in, but also as a virtue and a force that motivates and demands an active commitment to the other and compassion for the situation in which they find themselves.

**Table 1:** Training program on spirituality and hope in palliative care.

**Modules**	**Topics**	**Overview**
Module 1	Introduction to spirituality and hope in palliative care	Overview of the need to promote spirituality and hope in palliative care. Introduction to the basic terminology and principles of palliative care, with an emphasis on the biopsychosocial and spiritual domains. Analysis of topics related with the dynamics of hope, hopelessness, false hope, false hopelessness, and miracles and how they affect patient/family care (Marchand and Ingram, 2015; Olsman et al., 2016).
Module 2	The Professional as a tool of hope	The role of the professional as a tool to promote hope and the use of self in the therapeutic encounter. The professional's cosmovision and the characteristics of the hope encounter. Focus on self-awareness and self-consciousness by facilitating self-reflection on ones' spiritual needs, thoughts, beliefs, and values (Gijsberts et al., 2019; Chahrour et al., 2021) is paramount for ethical spiritual care. The therapeutic relation, compassionate care presence and hope communication strategies (Benito and Mindeguia, 2021).
Module 3	Cultural and spiritual issues	Overview of the cultural and spiritual dimensions in palliative care. Spiritual evaluations as necessary tools for effective communication and culturally appropriate care. For example, in certain cultures, despair is more closely associated with fatalism (an acceptance not accompanied by inactivity or helplessness), whereas optimism is expressed as confidence in a higher power absolving from duties for the consequences of illness (Merluzzi and Philip, 2017; Salamanca-Balen et al., 2021)
Module 4	Assessing hope and spirituality	Available tools to assess spirituality/hope in order to facilitate spiritual care in Palliative Care (Monod et al., 2011). Hope resources and symbols as an effective way to foster hope in palliative care (Querido, 2020; Querido et al., 2021). Hope communication tools (Olsman et al., 2015).
Module 5	Strategies to foster-hope	Brief self-reflective activities, arts and crafts, mindfulness in practice, appreciative inquiry, writing, collage, and other storytelling and debriefing strategies as tools to foster hope in patients and practitioners (Marchand and Ingram, 2015; Balboni et al., 2017; Laranjeira et al., 2020; Salamanca-Balen et al., 2021).
Module 6	Strategies fostering wellbeing, life review, and resource-based approach	Focus on Revie⊕: a life-review intervention based on a psychological model of dignity to improve the well-being of patients facing a life-limiting disease (Da Rocha Rodrigues et al., 2019). This topic reinforces the relevance of genuine presence and emphasizes the patient's personal resources when facing threatening experiences and regaining meaning in life and finitude.
Module 7	Hope legacy, farewell rituals and rites of passage	The legacy to others, meaning, gratitude, forgiveness, social support and relationship with others, and leaving something of value behind. Hope as a facilitator of anticipatory and preparatory grief. Farewell rituals/ rites of passage and grieving processes after a disaster or other traumatic event (Laranjeira and Querido, 2021).
Module 8	Translating evidence into practice	Hope-foster projects and their application in clinical practice—evaluation of the course by planning a hope intervention program in palliative settings.

## Final Remarks

Advocating for hope and spirituality in palliative care during the COVID-19 pandemic is critical because they help patients bring purpose to their lives. We should not remain inert in the face of difficulties, but should rather maintain hope and know-how to transform problems into experiences that promote our growth and learning. Spirituality and hope facilitate each other and together contribute to greater human wholeness by giving meaning and adjustment to negative life experiences.

## Author Contributions

All authors listed have made a substantial, direct, and intellectual contribution to the work and approved it for publication.

## Funding

This work is funded by national funds through FCT–Fundação para a Ciência e a Tecnologia, I. P. (UIDB/05704/2020 and UIDP/05704/2020) and under the Scientific Employment Stimulus—Institutional Call—(CEECINST/00051/2018).

## Conflict of Interest

The authors declare that the research was conducted in the absence of any commercial or financial relationships that could be construed as a potential conflict of interest.

## Publisher's Note

All claims expressed in this article are solely those of the authors and do not necessarily represent those of their affiliated organizations, or those of the publisher, the editors and the reviewers. Any product that may be evaluated in this article, or claim that may be made by its manufacturer, is not guaranteed or endorsed by the publisher.
